# Correlation between dynamic recrystallization and formation of rare earth texture in a Mg-Zn-Gd magnesium alloy during extrusion

**DOI:** 10.1038/s41598-018-35170-4

**Published:** 2018-11-14

**Authors:** M. G. Jiang, C. Xu, H. Yan, S. H. Lu, T. Nakata, C. S. Lao, R. S. Chen, S. Kamado, E. H. Han

**Affiliations:** 1 College of Mechatronics and Control Engineering, Shenzhen University, 3688 Nanhai Ave, Shenzhen, 518060 China; 2 The Group of Magnesium Alloys and Their Applications, Institute of Metal Research, Chinese Academy of Sciences, 62 Wencui Road, Shenyang, 110016 China; 3 School of Materials Science and Engineering, Harbin Institute of Technology, Harbin, 150001 China; 4 Department of Mechanical Engineering, Nagaoka University of Technology, Nagaoka, 940-2188 Japan

## Abstract

The trace addition of rare earth (RE) elements in Mg alloys can modify the extrusion texture, leading to the formation of RE texture and thus improved formability. The interrupted extrusion experiment as well as electron back-scatter diffraction (EBSD) characterization was conducted in Mg-1.5Zn-0.5Gd (wt.%) alloy to unveil the dominant dynamic recrystallization (DRX) mechanism and its correlation with the formation of RE texture during extrusion. The results indicate that continuous DRX (CDRX) dominated the microstructural development. Fresh DRXed grains with 30° [0001] grain boundaries preferentially nucleated in unDRXed grains with [10$$\bar{{\bf{1}}}$$0] basal fiber orientation via CDRX, showing preferred selection of [2$$\bar{{\bf{1}}}$$$$\bar{{\bf{1}}}$$0] basal fiber orientation rather than RE texture orientation. Consequently, CDRX contributed to the weakening of [10$$\bar{{\bf{1}}}$$0] basal fiber texture and had a more significant effect on the formation of [2$$\bar{{\bf{1}}}$$$$\bar{{\bf{1}}}$$0] basal fiber component than that of RE texture component. Besides, the preferred growth of recrystallized grains with RE texture orientation was confirmed to occur during static annealing after extrusion, which is inferred as the key reason for the formation of RE texture in dilute Mg-RE alloys.

## Introduction

Commercial Mg alloys usually develop a strong basal fiber texture during hot extrusion process, which accounts for the unsatisfactory formability and ductility at room temperature. The trace addition of rare earth (RE) elements has been recently claimed as an effective approach to modify the extrusion texture of Mg alloys, leading to the formation of a RE texture component with $< 11\bar{2}1 >$ directions parallel to the extrusion direction (ED)^1–5^, which contributes to significant improvement in ductility along the ED^1,2,4^.

Mg alloys are usually subjected to hot deformation for improving workability, during which dynamic recrystallization (DRX) often takes place. The final microstructure and mechanical properties of the alloys are closely related to the evolution of DRX process. There are two mechanisms mainly proposed that account for DRX in Mg alloy^6–9^: continuous DRX (CDRX) and discontinuous DRX (DDRX). The CDRX is featured by the development of low angle grain boundaries (LAGBs) and their progressive rotation into high angle grain boundaries (HAGBs), and thus new grains, which is different from the DDRX involving classic nucleation and subsequent growth of new DRXed grains. To date, the research on DRX behavior and its effect on texture formation during extrusion in RE-free Mg alloys has been well established^10–12^, but this is not the case for dilute Mg-RE alloys.

Some recent studies have already been devoted to the issue on the correlation with DRX and formation of RE texture in dilute Mg-RE extrusions^13–17^. Hadorn *et al*.^13,14^ and Robson^15^ hypothesized that CDRX is the key reason for the texture weakening and consequential formation of RE texture in dilute Mg-RE alloy during extrusion based on the evidence of RE solute segregation at GBs that is expected to hinder the motion of GBs and thus suppress the conventional DDRX process, but the authors did not further evidence experimentally whether the CDRX occurs and how such DRX mechanism affects the texture development during extrusion. Recently, Imandoust *et al*.^16^ studied the effect of RE element on the recrystallization textures in Mg-Ce and Mg-Gd binary alloys and reported that CDRX facilitated the transformation of a sharp <10$\bar{1}$0> basal fiber texture into a randomized texture. In contrast, their following study^17^ in the extruded Mg-Zn-Al-Y-MM (MM: Mischmetal) alloys revealed that CDRX sharpened the <10$\bar{1}$0> basal fiber texture and DDRX was shown to be the predominant mechanism for texture modification. Despite these efforts, there are still some differences of views on the DRX behavior in dilute Mg-RE alloys during extrusion and its corresponding contribution to the texture development. Besides, it should be noted that interrupted extrusion experiment is necessary to unveil the DRX behavior during extrusion, as this method is effective to reflect actual microstructural evolution and avoid the interference of DRXed grain growth during extrusion^10–12,18,19^, which was not adopted in Imandoust *et al*.’s studies^16,17^.

In our previous studies^4,20–22^, a series of Mg-Zn-Gd alloys have been developed as high-ductile wrought Mg alloys and it is found that a RE texture component formed in Mg-1.5Zn-0.5Gd (wt.%) alloy after extrusion under appropriate conditions^4^. In this study, therefore, interrupted extrusion experiment was designed for this Mg-Zn-Gd alloy, and electron back-scatter diffraction (EBSD) characterization was conducted to disclose the dominant DRX mechanism during extrusion and achieve a comprehensive understanding of the correlation between the DRX and the formation of RE texture for providing insightful knowledge into tailoring the texture formation and thus better designing new wrought Mg-RE alloys with high performance.

## Results

### Microstructure and RE texture of the extruded alloy

**Figure 1:**
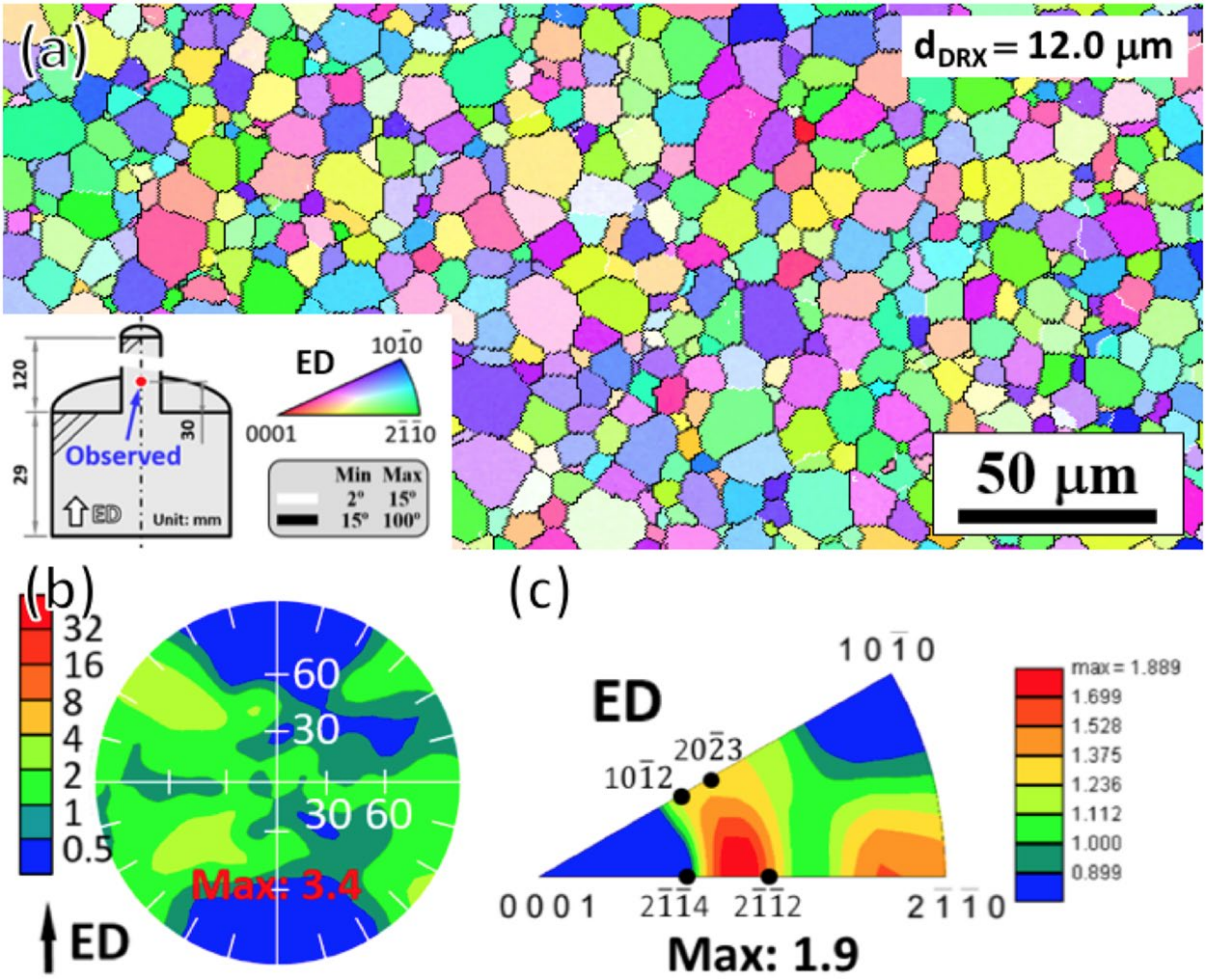
Microstructure and RE texture of the extruded alloy: (**a**) inverse pole figure map, (**b**) (0001) pole figure and (**c**) inverse pole figure.

The inverse pole figure map (Fig. 1a) shows a fully recrystallized microstructure with DRXed grain size of 12.0 μm. The (0001) pole figure (Fig. 1b) exhibits a weak extrusion texture with maximum intensity of 3.4 and basal poles showing a large angle distribution of ±60° away from the transverse direction (TD) to the ED (TD indicates the direction perpendicular to the ED). In the corresponding inverse pole figure (Fig. 1c), two texture components were evident: one was a [2$$\bar{1}$$$$\bar{1}$$0] basal fiber component with basal planes and [2$$\bar{1}$$$$\bar{1}$$0] directions parallel to the ED and the other was a non-basal component locating between [2$$\bar{1}$$$$\bar{1}$$4]//ED and [2$$\bar{1}$$$$\bar{1}$$2]//ED, i.e., RE texture component. This suggests that the RE texture component had already formed just after passage through the die.

### Microstructural evolution during extrusion

**Figure 2:**
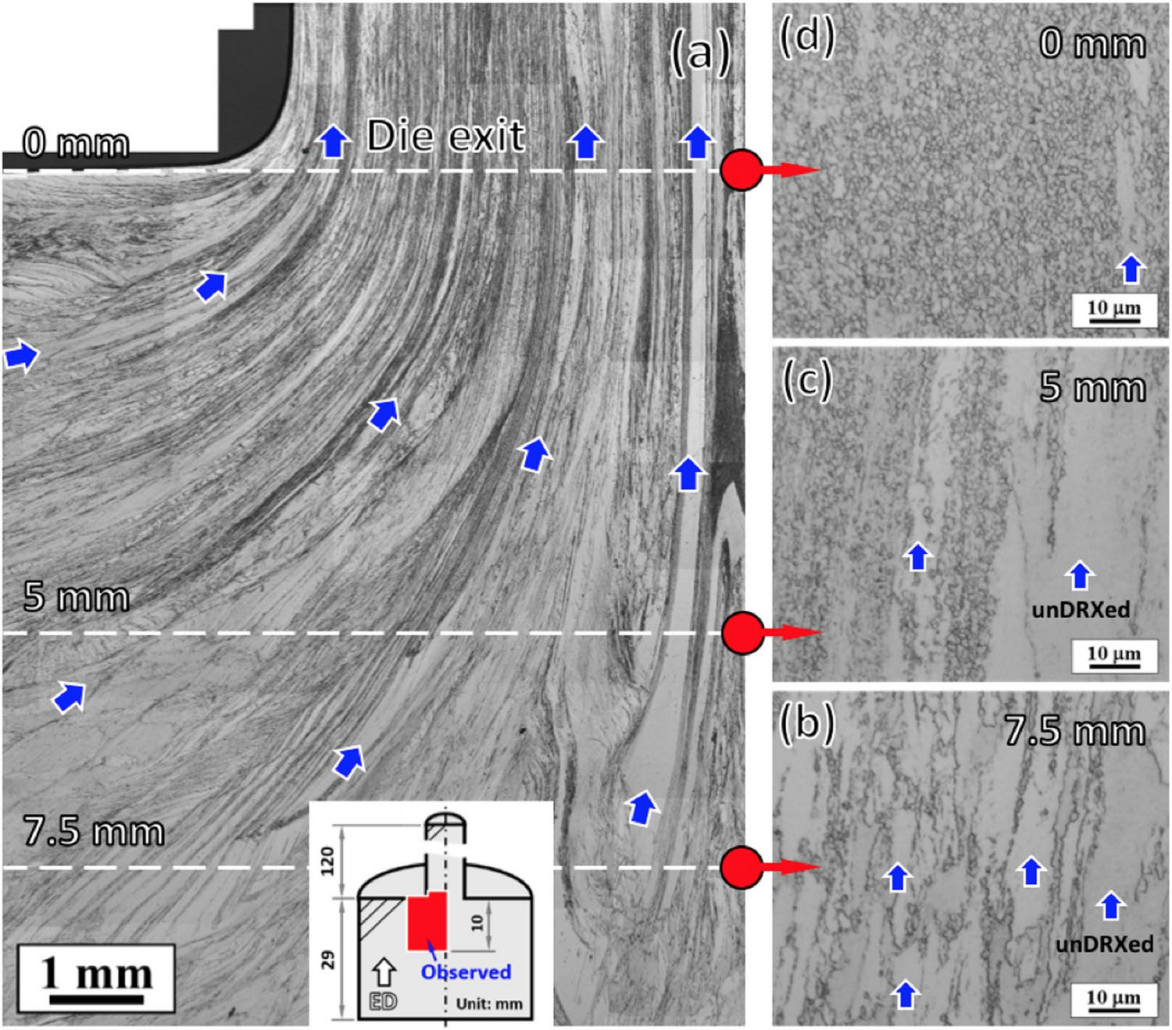
Optical microstructures of the interrupted extrusion sample at different locations shown in schematic illustration: (**a**) large area near the die exit and (**b**–**d**) indicated locations below the die exit. Blue arrows indicate the coarse unDRXed regions.

Figure 2 shows the optical microstructures of the interrupted extrusion sample at different locations below the die exit reflecting the microstructural evolution during extrusion. As shown in Fig. 2a, the microstructure showed a gradual change along the material flow toward the die exit. Besides, it is evident that very coarse unDRXed regions, as indicated by blue arrows, were elongated along the material flow and still existed after passage through the die. From the high magnified microstructures (Fig. 2b–d), a typical bimodal microstructure can be clearly observed, which consisted of coarse unDRXed and fine DRXed grains. At 7.5 mm (Fig. 2b), small DRXed grains (<1 μm) were observed with very low DRX faction, indicating the onset of DRX process. With further deformation (Fig. 2c,d), the DRX fraction increased with almost the same DRXed grain size. These observations suggest that the microstructure evolved as a consequence of DRX process in Mg-Zn-Gd alloy with negligible effect of DRXed grain growth during extrusion.

### DRX mechanisms and its effect on the texture development during extrusion

**Figure 3:**
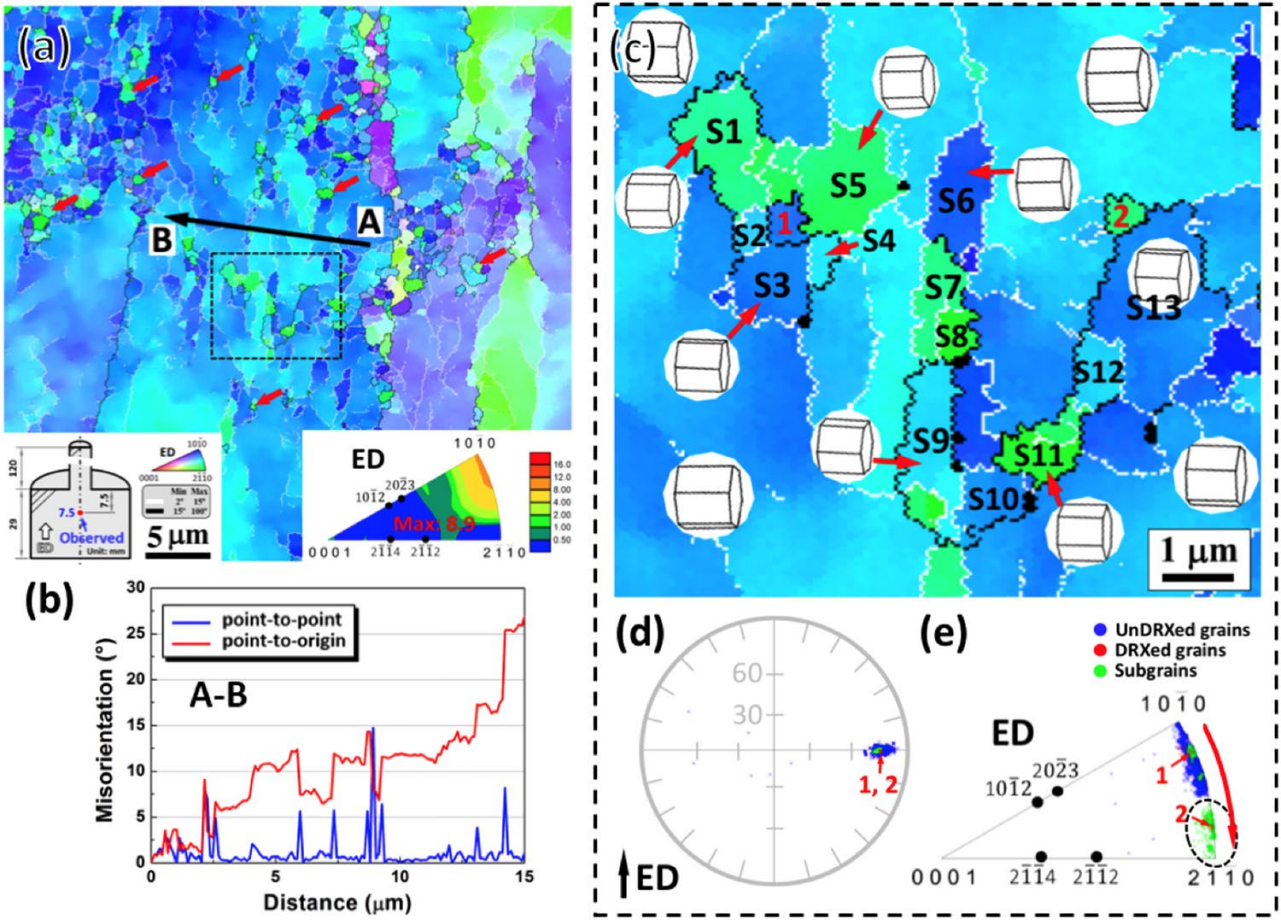
EBSD results of the interrupted extrusion sample at 7.5 mm below the die exit: (**a**) inverse pole figure map and corresponding inverse pole figure, (**b**) line profiles of misorientation angle along the direction indicated by black arrow AB in (**a**,**c**) enlarged inverse pole figure map in rectangular region shown in (**a**) and corresponding crystallographic orientations presented in (**d**) (0001) pole figure and (**e**) inverse pole figure. (S: subgrain, i = 1, 2…: DRXed grain).

Figure 3 shows the EBSD results of the interrupted extrusion sample at 7.5 mm below the die exit. Figure 3a shows that many fine DRXed grains with size of ∼1 μm formed in the elongated unDRXed grains as marked by red arrows. Besides, within the unDRXed grains there existed lots of LAGBs, which are usually related to the accumulation of dislocations in deformed structures^23,24^. The inverse pole figure inserted in Fig. 3a shows a strong [10$$\bar{1}$$0] basal fiber texture with maximum intensity of 8.9, in which the basal planes and [10$$\bar{1}$$0] directions aligned parallel to the ED. Figure 3b shows the line profile of misorientation angle along the direction indicated by black arrow AB in Fig. 3a. It is evident from point-to-origin that the misorientation angle raised up to ∼25°, suggesting the gradual rotation of orientation in the unDRXed grain. To clearly observe the formation of subgrains in the unDRXed grains and corresponding orientation relationship, a typical region comprising many subgrains as well as fine DRXed grains in Fig. 3a was selected as presented in Fig. 3c–e. Many subgrains bounded by LAGBs were observed as labelled by S1∼S13 and some sub-GBs had turned into HAGBs (Fig. 3c). It is inferred that more mobile dislocations would activate and accumulate in the vicinity of these LAGBs, and tend to transform into HAGBs with increasing strain, finally converting into fresh DRXed grains, which is classified as typical CDRX mechanism. As expected, fine DRXed grains of ∼0.5 μm labelled as 1 and 2 were observed in Fig. 3c. The corresponding crystallographic orientations (Fig. 3d,e) reveal that the subgrains and DRXed grains had very close basal plane orientations to their parent grains with basal planes aligning parallel to the ED (Fig. 3d), while the subgrains clustered at [2$$\bar{1}$$$$\bar{1}$$0]//ED with an evident deviation from their parent grains clustering at [10$$\bar{1}$$0]//ED (Fig. 3e).

**Figure 4:**
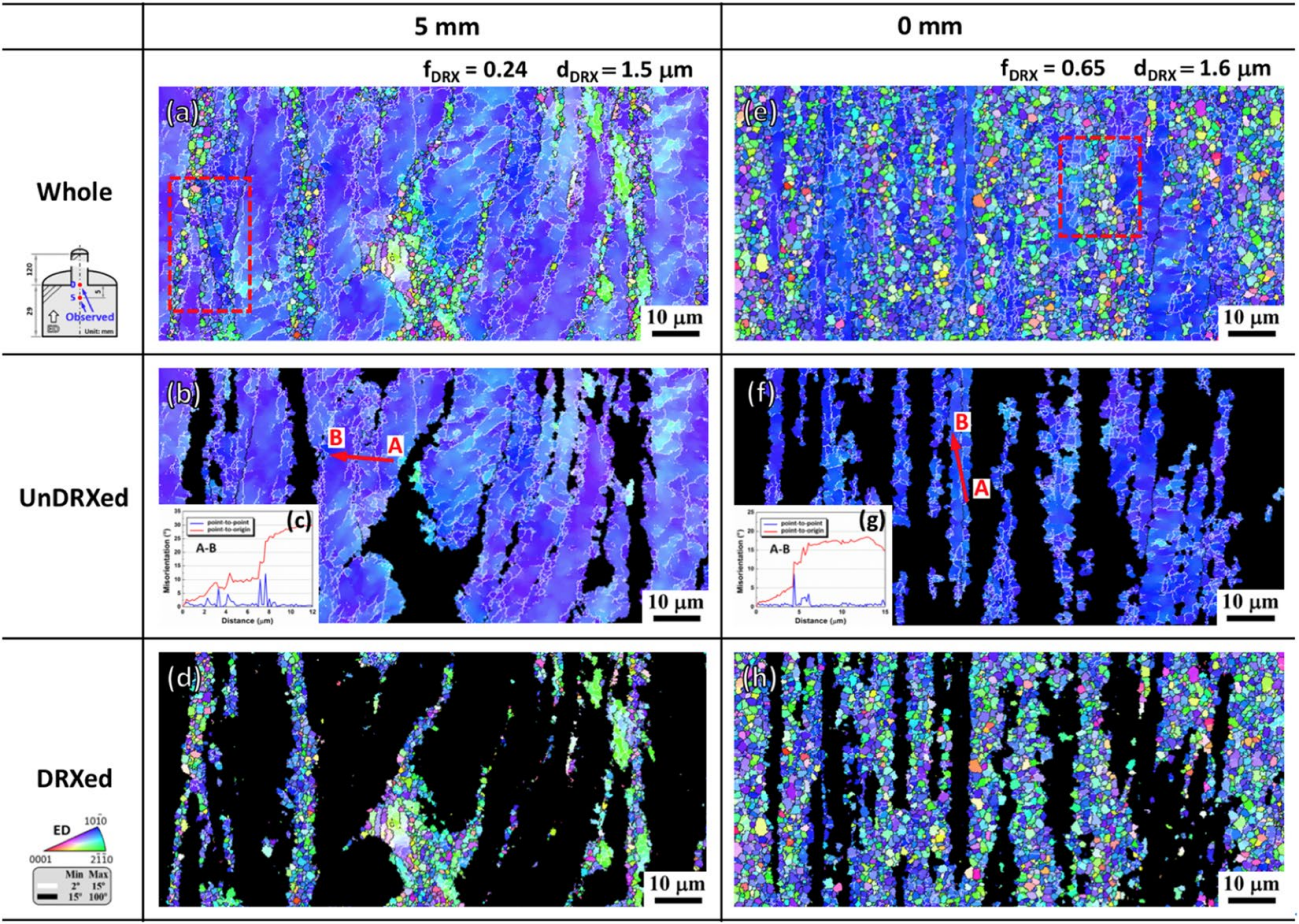
EBSD results of the interrupted extrusion sample at (**a**–**d**) 5 mm and (**e**–**h**) 0 mm below the die exit: (**a**,**b**,**d**–**f**,**h**) inverse pole figure maps of the whole, unDRXed and DRXed regions and (**c**,**g**) line profiles of misorientation angle along the direction indicated by red arrow AB in (**b**) and (**f**), respectively.

Figure 4 shows the EBSD results of the interrupted extrusion sample at 5 mm and 0 mm below the die exit. The inverse pole figures (Fig. 4a,e) exhibits a bimodal microstructure, as observed in Fig. 2, which that consisted of coarse unDRXed grains elongated along the ED and fine DRXed grains. With increasing strain, the DRX fraction increased drastically from ∼0.24 to ∼0.65, while the DRXed grains nearly stayed the same size (∼1.5 μm). Besides, high density of LAGBs were observed to form in the unDRXed grains (Fig. 4b,f). The line profiles of point-to-origin along the direction indicated by red arrow AB present a large increase in misorientation angle (Fig. 4c,g), indicating a gradual change of grain orientation due to the pile-up of dislocations. The above observations provide sound evidences of the occurrence of CDRX with high activity of LAGBs^8,9^.

**Figure 5:**
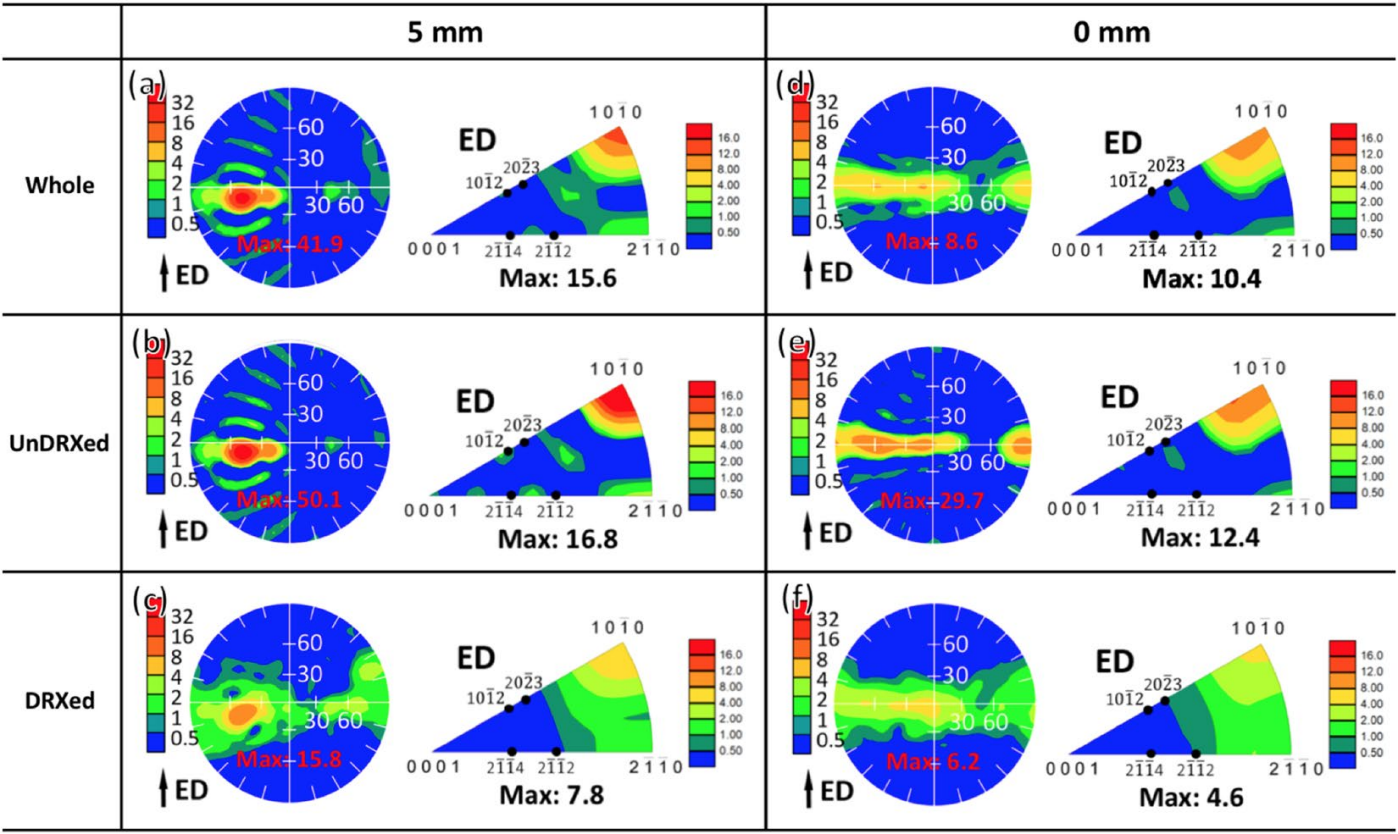
(0001) pole figures and corresponding inverse pole figures of the interrupted extrusion sample at (**a**–**c**) 5 mm and (**d**–**f**) 0 mm: (**a**,**d**) whole, (**b**,**e**) unDRXed and (**c**,**f**) DRXed regions.

**Figure 6:**
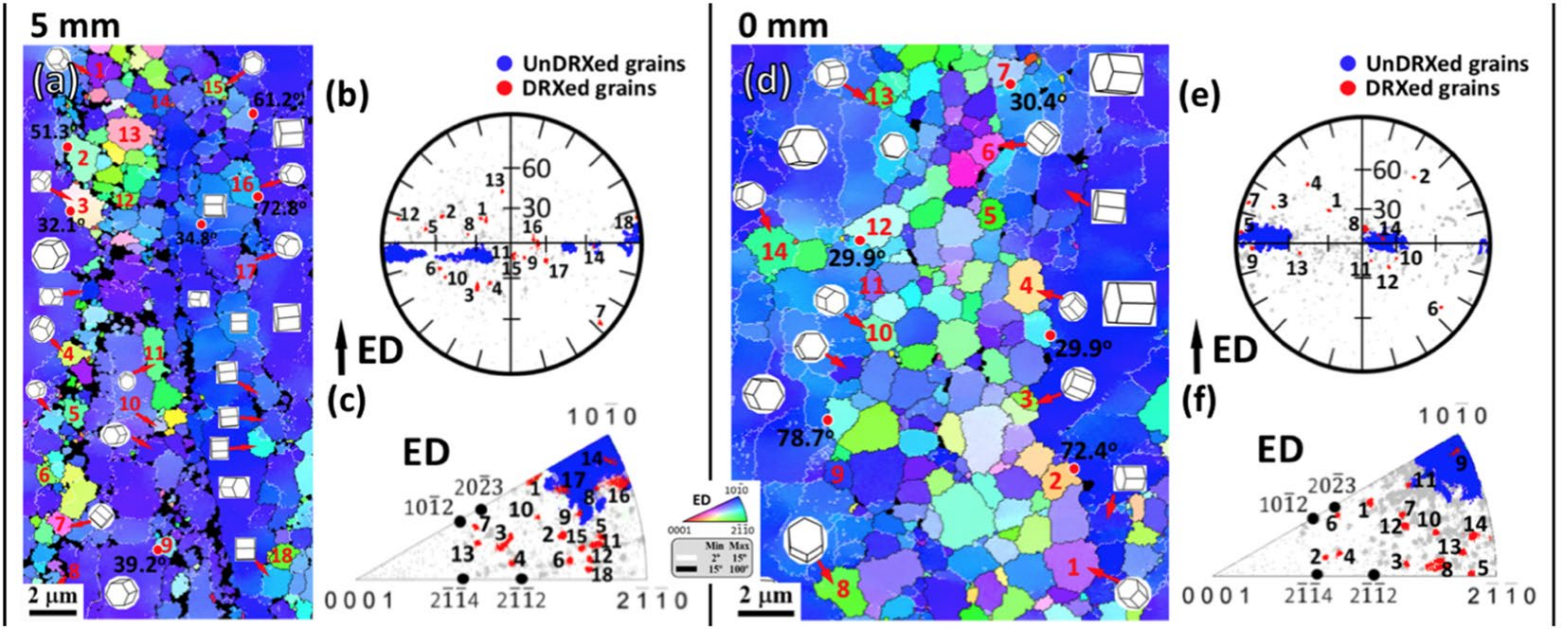
The crystallographic relationship between unDRXed and DRXed grains in the typical rectangular region shown in Fig. 4a,e: (**a**,**d**) inverse pole figure maps and corresponding crystallographic orientations presented in (**b**,**e**) (0001) pole figure and (**c**,**f**) inverse pole figure. Here, the light gray points in (**b**,**c**) and (**e**,**f**) indicate the orientations of the whole examined grains in (**a**,**d**). (i = 1, 2…: DRXed grain).

**Figure 7:**
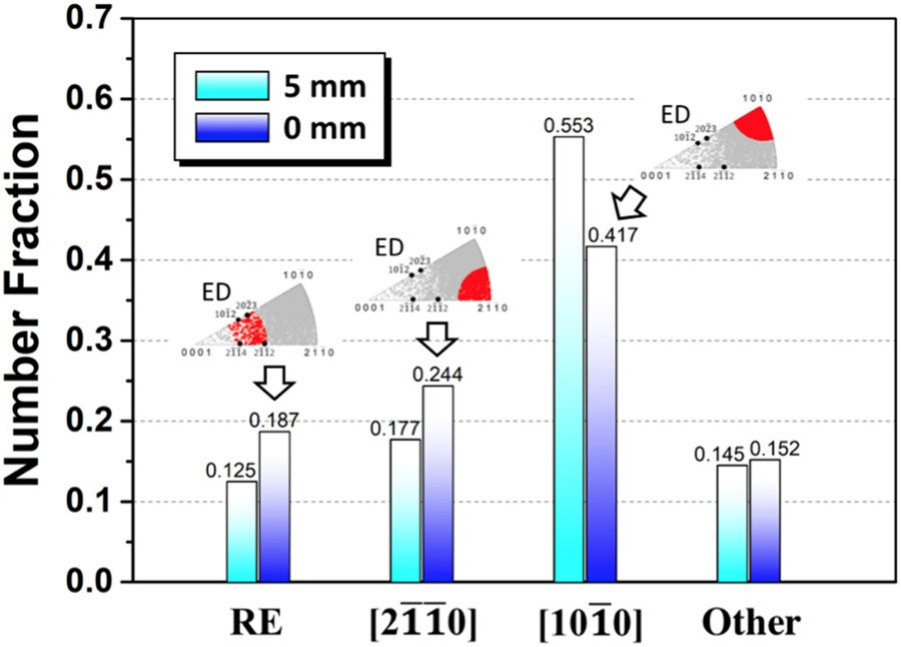
Statistical analysis of the fraction of DRXed grains with various orientations. For 0 mm, inverse pole figures are inserted, in which the light gray points indicate the orientations of the whole examined DRXed grains and highlighted red points indicate the selected orientations. (RE: RE texture orientation, [2$$\bar{1}$$$$\bar{1}$$0]: [2$$\bar{1}$$$$\bar{1}$$0] basal fiber orientation, [10$$\bar{1}$$0]: [10$$\bar{1}$$0] basal fiber orientation).

As shown in Fig. 5a,d, strong [10$$\bar{1}$$0] basal fiber texture formed with weak [2$$\bar{1}$$$$\bar{1}$$0] basal fiber component. Besides, it can be observed from 5 mm to 0 mm that [10$$\bar{1}$$0] basal fiber component became weakened with maximum texture intensity decreasing from 15.6 to 10.4 and [2$$\bar{1}$$$$\bar{1}$$0] basal fiber component stayed almost constant. To identify the contribution of CDRX process to the texture development, the (0001) pole figures and corresponding inverse pole figures of the unDRXed and DRXed regions were analyzed separately and presented in Fig. 5. Clearly, the unDRXed grains exhibited strong [10$$\bar{1}$$0] basal fiber texture (Fig. 5b,e), while the DRXed grains contributed to the weakening of [10$$\bar{1}$$0] basal fiber component and facilitated the formation of [2$$\bar{1}$$$$\bar{1}$$0] basal fiber component (Fig. 5c,f). It is beyond our expectation that RE texture component between [2$$\bar{1}$$$$\bar{1}$$4]//ED and [2$$\bar{1}$$$$\bar{1}$$2]//ED was completely absent. In order to further reveal the crystallographic relationship between unDRXed and DRXed grains, typical rectangular region was extracted from Fig. 4a,e, respectively, and some DRXed grains were randomly selected for analysis as presented in Fig. 6. The unDRXed grains showed [10$$\bar{1}$$0] basal fiber orientation, which is consistent with the observation in Fig. 5. Comparatively, the DRXed grains exhibited a wide spectrum of orientations, obviously deviating from their parent grains (unDRXed grains), but with a more preferred selection of basal texture orientation than RE texture orientation. For example (Fig. 6a–c), only DRXed grains 3, 4, 7 and 13 belonged to RE texture orientation (number fraction 4/18 = 0.22) and the others all belonged to basal texture orientation (number fraction 14/18 = 0.78). To make it more persuasive, more than 2000 DRXed grains observed at 5 mm and 0 mm were analyzed by EBSD, and the statistical results of the fraction of DRXed grains with various orientations are presented in Fig. 7. It can be observed that the total fraction of [10$$\bar{1}$$0] and [2$$\bar{1}$$$$\bar{1}$$0] basal fiber orientation was up to 0.730 and 0.661 at 5 mm and 0 mm, respectively, which is much higher than that of RE texture orientation (less than 0.2). As extrusion progressed from 5 mm to 0 mm, the fraction of [10$$\bar{1}$$0] basal fiber orientation dropped drastically and that of RE texture and [2$$\bar{1}$$$$\bar{1}$$0] basal fiber orientation exhibited a rising tendency. It is noted that the fraction of [2$$\bar{1}$$$$\bar{1}$$0] basal fiber orientation was ∼30% higher than that of RE texture orientation. Therefore, the above results point to the fact that CDRX contributed to the weakening of [10$$\bar{1}$$0] basal fiber texture and had a more significant effect on formation of [2$$\bar{1}$$$$\bar{1}$$0] basal fiber component than that of RE texture component.

## Discussion

**Figure 8:**
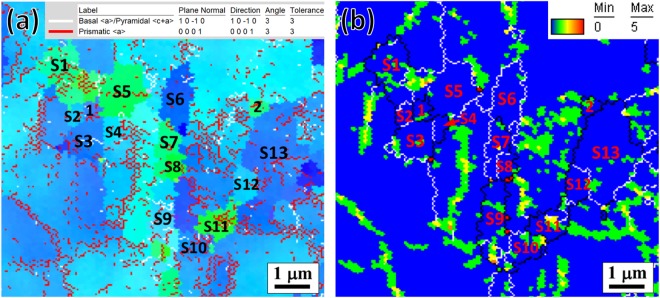
EBSD results of the typical rectangular region shown in Fig. 3a (at 7.5 mm): (**a**) inverse pole figure map showing the construction of LAGBs by arrays of basal <a>/pyramidal <c + a> and prismatic <a> slip dislocations and (**b**) corresponding KAM map showing the higher degrees of misorientation at the sites of the LAGBs. The white and red lines in (**a**) indicate the misorientations corresponding to the rotations around Taylor axes of basal <a>/pyramidal <c+a> and prismatic <a> dislocations, respectively. (S: subgrain, i = 1, 2…: DRXed grain).

In the current study, detailed EBSD observation provided direct evidence that CDRX dominated the microstructural development by the progressive *in-situ* rotation of subgrains in Mg-Zn-Gd alloy during extrusion with specific contribution to the alteration of extrusion texture. As CDRX is cross-slip of dislocations with <a> Burgers vector on non-basal planes^6,9^, intragranular misorientation axes (IGMA) analysis^13,16,25^ was applied to determine the activity of dislocations at the onset of CDRX (at 7.5 mm) as provided in Fig. 8a. The white and red lines indicate the misorientations corresponding to the rotations around Taylor axes of basal <a>/pyramidal <c + a> and prismatic <a> slip dislocations, respectively. The IGMA result reveals the predominant activity of prismatic <a> slip dislocations within and around the fresh subgrains. The corresponding kernel average misorientation (KAM) map (Fig. 8b), which is used to signify the stored strain energy (i.e., dislocation density) within materials^3,26^, confirms that such dislocation operation sites accompanied with higher local misorientation, giving rise to the progressive rotation of subgrains from parent orientation. Generally, non-basal slip systems operate more readily at high temperatures, as their CRSS values diminish markedly with increasing temperature^25,27,28^. According to the previous studies^25,29^, the CRSS value for pyramidal <c + a> slip is around 40 MPa at the extrusion temperature of 300 °C in this case, which is two times higher than that for prismatic <a> slip and basal slip. Besides, the addition of RE elements in Mg alloys could promote the activity of non-basal slip dislocations during ambient or high temperature deformation^13,30^. Form the above discussion, it is inferred that prismatic <a> slip dislocations operate more favorably during CDRX to rearrange themselves into cell structure or sub-GBs by cross-slip and climb.

**Figure 9:**
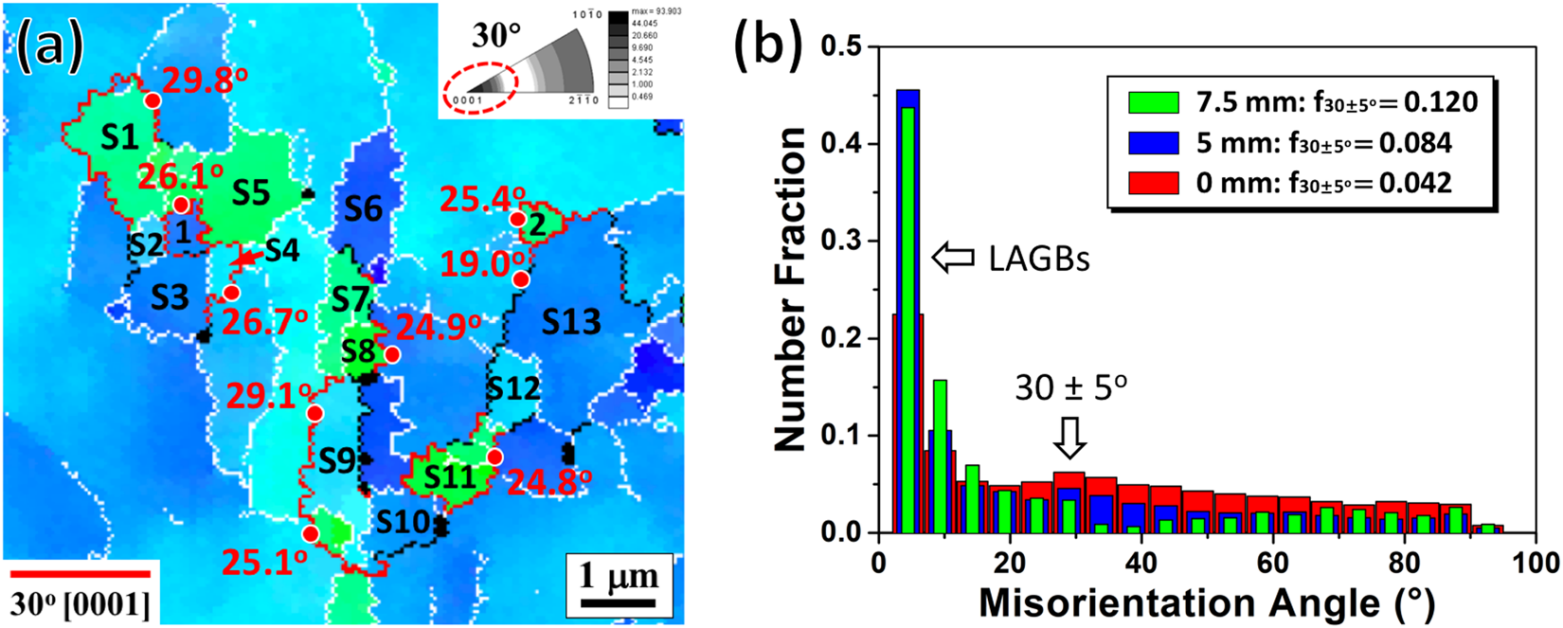
(**a**) Inverse pole figure map of the typical rectangular region in Fig. 3a (at 7.5 mm) with highlighted 30° [0001] GBs and (**b**) misorientation angle distributions showing the observed dominance of 30 ± 5° misorientation at different locations below the die exit. (S: subgrain, i = 1, 2…: DRXed grain).

In order to elucidate the effect of CDRX on the nucleation of new orientation, typical rectangular region shown in Fig. 3a was further analyzed as presented in Fig. 9a. Interestingly, most HAGBs of subgrains exhibited a similar GB type with misorientation of 30 ± 5° around [0001] axis and the newly formed DRXed grains 1 and 2 were bounded by these special GBs as highlighted in red. This significance of 30° [0001] GBs can be further confirmed from the rotation axis distribution in Fig. 9a. The misorientation angle distributions (Fig. 9b) display the distinct number fraction for LAGBs and another local peak at 30 ± 5°. It is noted that the peak at 30° became evident gradually and the corresponding number fraction increased from 0.042 to 0.120 with increasing strain. This well supports the observed dominance of 30° [0001] GBs during extrusion, which from the orientation perspective is responsible for the nucleation of new [2$$\bar{1}$$$$\bar{1}$$0] basal fiber orientation in the unDRXed grains via CDRX. The 30° [0001] GBs have been often observed in the recrystallized microstructures of Mg alloys mainly ascribed to the formation of ∑13a coincident site lattice (CSL) boundaries^12,31–35^. Molecular dynamic simulations^34,35^ disclosed that such GBs possessed both low interfacial energy and high mobility relative to other boundaries, making them prime candidates for accommodating subgrain rotation.

It has been evidenced by the researchers^14,36–39^ that RE solutes segregate strongly to GBs and thus affect the interfacial mobility through solute drag effect caused by strong interactions between the RE solutes and the GBs, which is likely to alter the DRX mechanism and subsequent grain growth behavior. In contrast, non-RE elements such as Al and Zn with small atomic radius and low diffusion rate do not have such propensity^15,37^. Recently, Barrett *et al*.^40^ studied the effect of RE element segregation on the GB energy and mobility in Mg via a combined study of EBSD analysis and molecular dynamic simulations and proposed that RE element homogenized the GB energy and mobility variations among GB types, reducing the preferred selection of special 30° [0001] GBs for stabilization and growth and thus providing a more equal opportunity for other orientations to thrive. Despite the addition of RE element, however, the nucleation of DRXed grains with 30° [0001] GBs was still predominant with enhancement of [2$$\bar{1}$$$$\bar{1}$$0] basal fiber orientation in this study. According to the above Barrett *et al*.’s viewpoint^40^, this is most likely due to the insufficient RE segregation at GBs at the DRX nucleation stage in this case.

**Figure 10:**
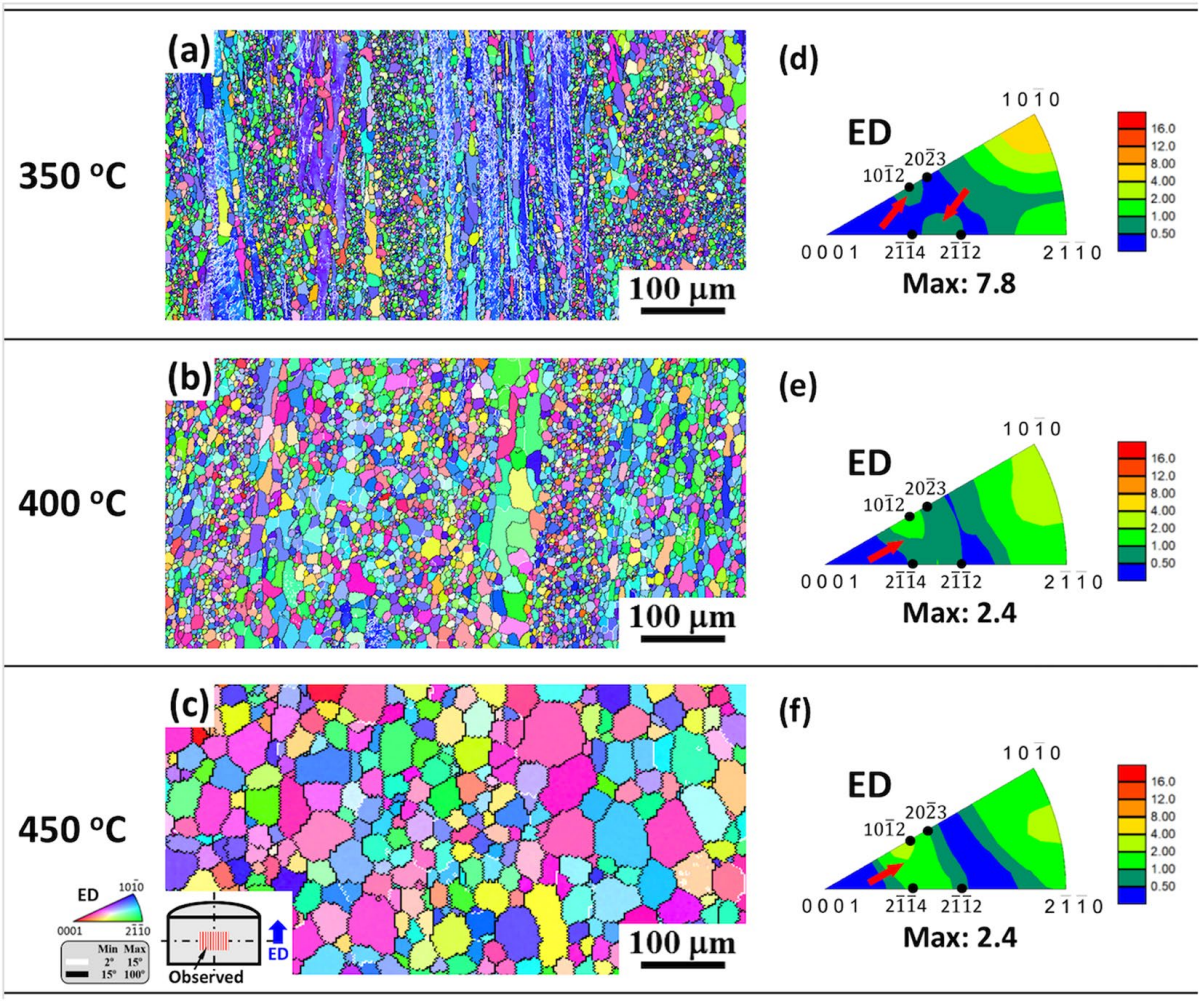
(**a**–**c**) Inverse pole figure maps and (**d**–**f**) corresponding inverse pole figures of the extruded alloy (processed at 350 °C and 1 mm/s) after annealing at various temperatures for 10 min: (**a**,**d**) 350 °C, (**b**,**e**) 400 °C and (**c**,**f**) 450 °C.

**Figure 11:**
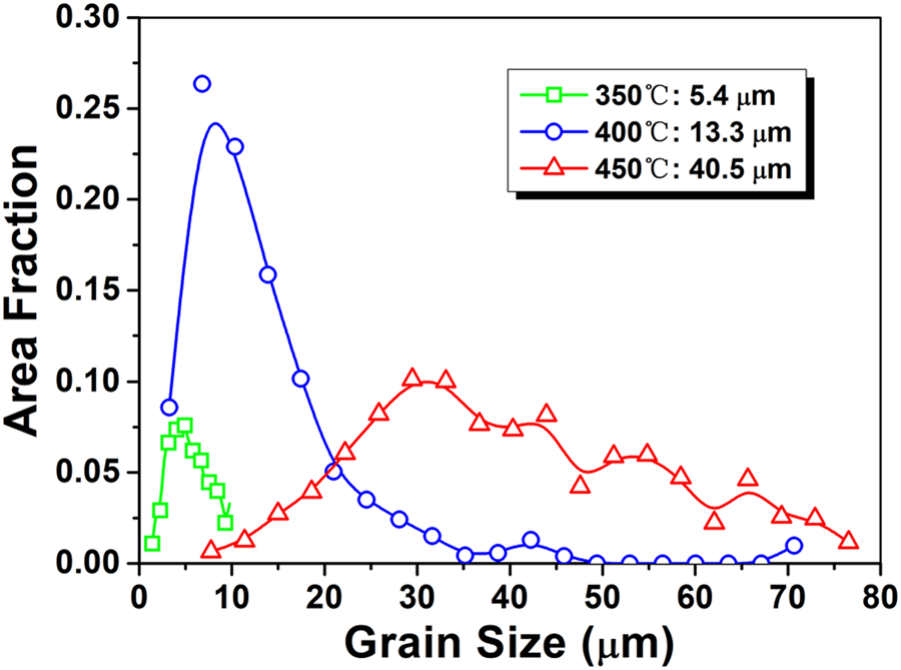
Recrystallized grain size distributions of the extruded alloy (processed at 350 °C and 1 mm/s) after annealing at various temperatures for 10 min.

Previous studies^36,38,39^ have revealed that RE elements have a larger tendency to segregate at GBs during grain growth upon annealing, since there is sufficient heat stimulation for RE solutes to diffuse from the grain interiors to the GBs. As a consequence, solute drag effect becomes strengthened during grain growth, which is expected to hinder the mobility of 30° [0001] GBs and thus preferred growth of grains with [2$$\bar{1}$$$$\bar{1}$$0] basal fiber orientation. It should be noted that the material is subjected to the intense deformation and friction heating in the die and bearing surfaces during extrusion, especially under high extrusion speed, which makes static annealing inevitable after passing through the die despite the immediate water-quenching in this study. This is possible to result in the development of fully DRXed microstructure and RE texture component (Fig. 1). To confirm the preferred orientation selection during grain growth, the extruded Mg-Zn-Gd alloy having similar bimodal microstructure with that observed at the die exit for the interrupted extrusion sample (Fig. 4e) was prepared and then subjected to annealing treatment at various temperatures ranging from 350 °C to 450 °C for 10 min. The analyzed results are presented in Figs 10 and 11. The inverse pole figure maps (Fig. 10a–c) demonstrate the gradually increased recrystallized fraction and grain size with increasing annealing temperature and that the alloy annealed at 450 °C exhibited a fully recrystallized and homogeneous microstructure. As shown in Fig. 11, the DRXed grains grew gradually from 5.4 μm to 40.5 μm with increasing annealing temperature and meanwhile the recrystallized grain size distribution became wider. This further confirms that consecutive grain growth occurred during annealing. The corresponding inverse pole figures (Fig. 10d–f) clearly reveal that with increasing annealing temperature RE texture component formed gradually as indicated by red arrows and became intensified with the weakening of [10$$\bar{1}$$0] basal fiber component, which is indicative of the preferred growth of recrystallized grains with RE texture orientation during annealing.

It is known that the final recrystallization texture either depends on the oriented nucleation of new grains or the subsequent favorable growth of grains with specific orientations or both^41^. This study evidenced that the DRXed grains tended to possess the preferred selection of [2$$\bar{1}$$$$\bar{1}$$0] basal fiber orientation via CDRX during extrusion, which is consistent with the observations in pure Mg^31–33^ and Mg-Al alloys^12,35^. But the subsequent favorable growth of RE texture orientation in this case is quite different from that in RE-free Mg alloys, in which the grains with <11$$\bar{2}$$0> basal fiber orientation showed an overwhelming growth advantage over that with other orientations during annealing^42–44^. Recently, Zeng *et al*.^44^ provided convincing evidence of the preferred growth of grains with off-basal texture orientation in Mg-Zn-Ca alloy during static recrystallization using quasi-*in-situ* EBSD method and characterized the co-segregation of Ca and Zn at GBs using high-angle annular dark-field scanning transmission electron microscopy (HAADF-TEM). Based on these results, they further hypothesized that Ca and Zn solutes are prone to co-segregate at the GBs of grains with <11$$\bar{2}$$0> basal fiber orientation, reducing the mobility of such GBs by solute drag effect and thus contributing to the weakening of basal texture^44^. Nevertheless, it is difficult to prove whether the solute segregation occurs uniformly or only at some specific type of GBs such as 30° [0001] GBs and also very challenging to establish the quantitative relation between the RE solute concentration at GBs and the growth rate of such GBs, which needs further clarification in the future. What becomes clear in this study is that the subsequent favorable growth of RE texture orientation makes a much more significant contribution to the formation of RE texture rather than the DRX mechanism in dilute Mg-RE alloys during extrusion.

On the other hand, RE solute clustering within matrix has been expected as effective obstacles to the motion of dislocations through solute drag effect^14^. Specifically, sufficient RE solute clustering may interact with prismatic <a> slip dislocations that operate more readily in this case and promote cross-slip, thereby facilitating the occurrence of CDRX process. Additionally, DDRX mechanism is considered to be strongly suppressed by RE solute segregation at GBs^13–15^, since it requires higher mobility of GBs than CDRX. Hence, the microstructure evolved dominantly as a result of CDRX during extrusion in this study. It is noted that increased extrusion temperature is likely to facilitate the transition in DRX mode from CDRX to DDRX, because the velocity of dislocations as well as GBs becomes high enough to break free of any RE solutes atmosphere that develops at high temperatures, leading to a decreased solute drag effect^37^. This is also a plausible explanation for the disappearance of RE texture component at high extrusion temperatures (>490 °C)^1,37^. Experimental evidence indicates that RE addition is effective in suppressing the DRX progress that occurs during deformation at appropriate temperatures^16,45,46^, which is different from the DRX behavior in AZ31 alloy^11,12^. For dilute Mg-RE alloys, therefore, the suppressed DRX process enables a higher stored stain energy to be retained prior to static annealing for driving the subsequent DRXed grain growth.

In summary, we minimized the interference of DRXed grain growth and clearly unveiled the dominant DRX mechanism and its correlation with the formation of RE texture in the Mg-Zn-Gd alloy during extrusion using interrupted extrusion method combined with EBSD characterization. The extruded alloy showed a fully recrystallized microstructure with DRXed grain size of 12.0 μm and weak RE texture component between [2$$\bar{1}$$$$\bar{1}$$4]//ED and [2$$\bar{1}$$$$\bar{1}$$2]//ED. The observation of interrupted extrusion sample revealed that CDRX dominated the microstructural development during extrusion. Subgrains continuously nucleated in the unDRXed grains with [10$$\bar{1}$$0] basal fiber orientation mainly through rearrangement of prismatic <a> slip dislocations, gradually converting into fresh DRXed grains. These DRXed grains tended to possess 30° [0001] GBs and thus exhibited the preferred selection of [2$$\bar{1}$$$$\bar{1}$$0] basal fiber orientation. Consequently, CDRX contributed to the weakening of [10$$\bar{1}$$0] basal fiber texture and had a more significant effect on the formation of [2$$\bar{1}$$$$\bar{1}$$0] basal fiber component than that of RE texture component. Besides, the preferred growth of recrystallized grains with RE texture orientation was confirmed to occur during static annealing after extrusion, most likely due to the solute drag effect caused by RE solute segregation at GBs. This can be inferred as the key reason for the formation of RE texture in dilute Mg-RE alloys. These findings assist in understanding how RE element modify the extrusion texture in Mg alloys and thus better designing new wrought Mg-RE alloys with improved formability and ductility.

## Methods

### Material preparation

Alloy ingot with a composition of Mg-1.58Zn-0.52Gd (wt.%) was prepared by high purity 99.9% Mg, 99.9% Zn, 99.95% Gd by resistance melting under the protection of a gas mixture of SF_6_ (1 Vol.%) and CO_2_ (99 Vol.%). The ingot was homogenized at 480 °C for 12 h and quenched into hot water, and subsequently machined to cylindrical billets with a diameter of 43 mm and a height of 35 mm for extrusion. The indirect extrusion was carried out at 300 °C with a ram speed of 20 mm/s and an extrusion ratio of 20, and then interrupted when the material had emerged ∼120 mm from the die exit, and finally quenched into water with purpose of observing microstructure and texture evolution during extrusion. To confirm the effect of grain growth on texture development, the billet was extruded at 350 °C with a ram speed of 1 mm/s and an extrusion ratio of 20 to obtain typical bimodal microstructure as provided in our previous study^47^ and then subjected to annealing treatment at various temperatures ranging from 350 °C to 450 °C for 10 min, followed by water-quenching.

### Microstructure characterization

The microstructures were observed using optical microscope (OM, Olympus BX51M) and electron back-scatter diffraction (EBSD). The interrupted extrusion sample was sectioned along the central plane parallel to the ED and the microstructures were observed at various locations right below the die exit. Samples for OM observation were etched in a solution of acetic picral (2.2 g of picric acid, 2 ml of acetic acid, 35 ml of ethanol and 3 ml of water) after mechanical polishing. EBSD was conducted using JEOL JSM-7000F field-emission scanning electron microscope (FE-SEM) equipped with an EDAX-TSL EBSD system operating at 15 kV, and the data were analyzed by OIM Analysis software. The diffraction patterns have high average confidence index ranging from 0.44 to 0.66, suggesting the sound quality of EBSD results. For the interrupted extrusion sample, appropriate area containing more than 1000 grains was examined with step size of 0.13∼0.2 μm for texture analysis to guarantee the data reliability and some areas of interest (at 0 and 5 mm) were selected and examined with smaller step size of 0.06 μm for further detailed analysis. For the annealed samples, more than 2000 grains were examined with step size of 0.8∼4 μm for each condition. Image-Pro Plus 6.0 software was used to determine the DRX fraction (area fraction) based on the examined inverse pole figure maps.
